# Balloon Pulmonary Angioplasty in Patients with Chronic Thromboembolic Pulmonary Hypertension in Greece: Data from the Hellenic Pulmonary Hypertension Registry

**DOI:** 10.3390/jcm11082211

**Published:** 2022-04-15

**Authors:** Panagiotis Karyofyllis, Eftychia Demerouti, George Giannakoulas, Anastasia Anthi, Alexandra Arvanitaki, George Athanassopoulos, Christos Feloukidis, Ioannis Iakovou, Theodora Kostelidou, Ioanna Mitrouska, Sophia-Anastasia Mouratoglou, Stylianos E. Orfanos, Christos Pappas, Georgia Pitsiou, Eleftheria-Garyfallia Tsetika, Dimitrios Tsiapras, Vassilios Voudris, Athanassios Manginas

**Affiliations:** 1 Cardiology Department, Onassis Cardiac Surgery Center, 17674 Athens, Greece; pakar768@yahoo.gr (P.K.); george.d.athanassopoulos@gmail.com (G.A.); Ioannis.iakovou@gmail.com (I.I.); kostelidou@ocsc.gr (T.K.); elina_tse@live.com (E.-G.T.); dtsiapras@hotmail.com (D.T.); vvoudris@otenet.gr (V.V.); 2 Cardiology Department, AHEPA University Hospital, Aristotle University of Thessaloniki, 54621 Thessaloniki, Greece; g.giannakoulas@gmail.com (G.G.); alexandra.arvanit@gmail.com (A.A.); cfelouk@gmail.com (C.F.); s_mouratoglou@yahoo.gr (S.-A.M.); 3 1st Department of Critical Care, National & Kapodistrian University of Athens Medical School, Pulmonary Hypertension Clinic Evaggelismos General Hospital, 10676 Athens, Greece; anastasia.anthi1@gmail.com (A.A.); stylianosorfanosuoa@gmail.com (S.E.O.); 4 Department of Intensive Care Medicine, University Hospital of Heraklion, 71110 Heraklion, Greece; mitrouska@med.uoc.gr; 5 Multidisciplinary Pulmonary Hypertension Center, Attikon University General Hospital, 12461 Athens, Greece; clincard@hotmail.com; 6 Respiratory Failure Unit, “G. Papanikolaou” Hospital, 57010 Thessaloniki, Greece; gpitsiou@yahoo.gr; 7 Cardiology Department, Mediterraneo Hospital, 16675 Athens, Greece; nassoseft@yahoo.com

**Keywords:** chronic thromboembolic pulmonary hypertension, balloon pulmonary angioplasty, pulmonary hypertension, registry

## Abstract

Balloon pulmonary angioplasty (BPA) is a novel and promising treatment option for patients with chronic thromboembolic pulmonary hypertension (CTEPH) who are ineligible for pulmonary endarterectomy (PEA) and for those with persistent or recurrent pulmonary hypertension after PEA. We present the results of BPA procedures in CTEPH patients included in the Greek Pulmonary Hypertension Registry, evaluating the real-life efficacy and safety. We analyzed data from 180 BPA procedures (2–17/patient, mean 8 ± 4/patient, 1248 dilated vessels, 0–18/session). Significant improvements were observed in mean pulmonary arterial pressure (a reduction by 44%, *p* < 0.001), pulmonary vascular resistance (reduction by 60%, *p* < 0.001), and NT-proBNP (decrease by >70%, *p*: 0.003), while cardiac index improved modestly (9% increase, *p* = 0.143). We had 37 BPA-related non-fatal complications (20.6% in all interventions), predominantly including hemoptysis. Overall survival was 91%, 75% and 62% at 3, 4 and 5 years, respectively. Therefore, BPA may be a promising therapeutic option in patients with CTEPH in Greece.

## 1. Introduction

Chronic thromboembolic pulmonary hypertension (CTEPH) is characterized by organized thromboembolic material leading to increased pulmonary vascular resistance (PVR) in the pulmonary vascular bed. The prognosis is poor due to right ventricular failure and death [1]. According to the latest pulmonary hypertension (PH) guidelines [2], CTEPH is a precapillary PH with a mean pulmonary arterial pressure (mPAP) of more than 25 mmHg and a pulmonary arterial wedge pressure (PAWP) lower than 15 mmHg as measured by right heart catheterization (RHC). At the 6th World PH Symposium, a new definition was proposed [3] as the value of mPAP is lower at 20 mmHg, however pulmonary endarterectomy (PEA) remains the gold standard of treatment.

PEA is the treatment of choice for CTEPH patients. However, up to 51% develop persistent/recurrent PH after PEA [4], which is associated with persistent symptoms and a poor prognosis. Furthermore, many patients are deemed inoperable by expert surgeons due to more distal disease, comorbidities or an extremely high risk for surgery. Moreover, some patients refuse the surgical approach. In cases of inoperable or persistent CTEPH, balloon pulmonary angioplasty (BPA) represents an extremely promising option with many conducted studies demonstrating its efficacy and safety in improving symptoms, exercise tolerance, right heart function, and long-term prognosis. [5,6,7].

The aim of this study was to evaluate the real-life efficacy and safety of BPA in CTEPH patients enrolled in the Hellenic Pulmonary Hypertension Registry (HOPE registry).

## 2. Materials and Methods

The HOPE registry [8], launched in January 2015, enrolls patients mainly with pulmonary arterial hypertension (PAH) and CTEPH, is approved by the Institutional Review Board of each one of the ten participating PH expert centers in Greece according to the Declaration of Helsinki and all patients provided written informed consent for their enrolment. Documentation has been Internet-based (PAH tool by Inovultus Lda, Portugal). The cut-off day for the analysis provided in this article was 10 February 2022. 

CTEPH was defined by the following: (1) mPAP > 25 mmHg with PAWP ≤ 15 mmHg and PVR > 3 WU as hemodynamic parameters; (2) abnormal ventilation–perfusion (VQ) scanning, invasive pulmonary angiography, computed tomography pulmonary angiography or magnetic resonance pulmonary angiography confirming chronic thromboembolic obstructions as described in European Guidelines [2]. The hemodynamic definition used in our population is based on the latest guidelines for the diagnosis and treatment of PH [2] as the patients enrolled before the 6th World PH Symposium where a new definition was proposed [3].

Hemodynamic parameters were evaluated during a RHC as follows: right atrial mean pressure (RAP), pulmonary artery pressure (systolic, diastolic) and mean pulmonary arterial pressure (mPAP), pulmonary capillary wedge pressure (PAWP), pulmonary vascular resistance (PVR) and cardiac index (CI) with the Fick method, as thermodilution is not provided in the implicated centers; evaluation of the level of Natriuretic Peptides, N-terminal pro Brain Natriuretic Peptide (NT-proBNP) and the need for oxygen therapy are provided prior to and after the BPA procedure. All these parameters were endpoints of efficacy, measured prior to the first BPA (baseline data) and after the last BPA. For patients who had not completed the BPA therapy until the data cut off, the provided efficacy endpoints were collected during their last BPA session. Safety data were collected up to 10 February 2022. 

### 2.1. Patients’ Characteristics

In total, 27 adult patients with CTEPH were enrolled for BPA from January 2015 until February 2022. CTEPH diagnosis was established excluding other forms of PH according to the current guidelines [2]. Inclusion criteria were a diagnosis of CTEPH, age > 14 years, after at least three months of adequate anticoagulation therapy and available data from right heart catheterization (RHC) [9].

All patients were evaluated for eligibility for PEA. Six patients (22.2%) had already undergone PEA with increased risk of reoperation. Thirteen patients (48.1%) were deemed inoperable due to surgically inaccessible lesions and an unfavorable risk/benefit ratio based on comorbidities and older age; specifically, 9 (37.5%) due to subsegmental disease and 4 (16.7%) due to comorbidities and older age. The remaining 8 patients refused PEA. The assessment of operability was evaluated by an experienced team abroad due to the absence of a dedicated expert center for PEA in Greece. Due to missing data concerning follow up based on hemodynamic and biochemical parameters, 3 out of 27 patients were excluded for further analysis.

### 2.2. BPA Procedure

In total, we analyzed data provided until the cut-off date for this study. BPA took place in two expert PH centers in Greece by two interventional cardiologists with expertise in this procedure. Twenty-four patients were treated at the Onassis Cardiac Surgery Center (all data integrated in analysis) and the other 3 patients at Attikon Hospital; however, all necessary data were not provided, so these patients will not be included in this study for further analysis. The first BPA procedure took place on December 2016 at the Onassis Cardiac Surgery Center.

Right-heart catheterization was performed at the beginning of each procedure. Oral anticoagulation was not discontinued before the BPA sessions. An additional 500–2000 units of heparin were administered to reach an activated clotting time of around 200 s. Oxygen was administered to maintain an oxygen saturation of more than 98% and electrocardiogram (ECG), blood pressure and heart rate were monitored.

We predominantly used access via femoral vein. A jugular vein access was employed for only three procedures in one patient. At the beginning of each intervention, RHC was performed using an 8- or 9-French sheath and a Swan–Ganz catheter (Edwards Lifesciences). BPA was initiated and a 6- or 7-French sheath (70 cm or 90 cm depending on patient metrics) containing a pre-shaped 6-or 7-French guiding catheter (Multipurpose, Judkins Right-4, Amplatz Left-1, EBU 3.0; Medtronic, Minneapolis, MN, USA) was moved into the pulmonary artery using the 8- or 9-French sheath and a 0.035-in. hydrophilic wire.

**Figure 1:**
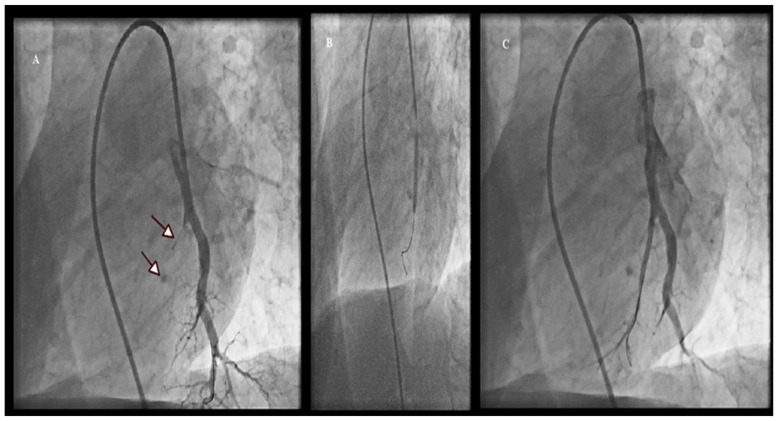
Selective pulmonary angiogram in the left lung (**A**) shows a total occluded subsegmental branch of LA9 (arrows), the inflation of a 3.0 × 20 mm balloon (**B**) and the final result after the BPA procedure (**C**).

In the initial session of each patient, the target vessels were selected and the lower lobe lesions were firstly dilated because the pulmonary blood flow at this region is higher, leading to a lower mPAP. A 0.014-inch guide wire (Asahi Sion Blue, Asahi Intecc Co., Japan or PT2 Moderate Support, Boston Scientific, Marlborough, MA, USA) was placed into the target branch during the initial sessions. When the procedure was not performed in the upper lobe, the pulmonary artery was stretched via a deep inhalation leading to an easier passage of the guidewire. We selected undersized balloons (Solarice 1.5–2/20 mm, Medtronic, Minneapolis, MN, USA) during the initial sessions of each patient to avoid pulmonary vascular injury. Larger balloons (3.0 mm to 9.0 mm), depending on vessel diameter, were used in subsequent sessions for post-dilatation. Multiple balloon inflations were performed at 2–17 segmental or subsegmental branches during a session. Other modalities, such as optical coherence tomography (OCT) and fractional flow reserve (FFR) were not used; however, intravascular ultrasound (IVUS) was performed in one patient with a chronic total occlusion. We limited each procedure to 300 mL of contrast medium or 60 min of total fluoroscopy time. A following procedure was planned within 1–8 weeks. Figure 1 demonstrates selective pulmonary angiograms and BPA result in an inoperable patient with CTEPH.

### 2.3. Definition of Complications Related to BPA

During the 6th world symposium on PH, the task force on CTEPH proposed a definition and classification of complications related to BPA [10]. According to this publication, vascular injury appears to be the most common complication and cause of lung injury (LI) characterized by lung opacities in chest X-ray or CT scan, associated or not with hemoptysis. The main underlying cause seems to be hemorrhage, complication due to distal vascular injury, which can be provoked by iatrogenic wire injury, over dilation of the lesion or high-pressure contrast injection. Vascular dissection, allergic reaction to contrast or to local anesthesia are also complications during the procedure [11,12,13]. After the procedure, renal dysfunction and access site problems also represent BPA complications.

In our study, complications related to BPA were defined as follows: Hemoptysis, asymptomatic LI defined as opacities on chest X-ray and/or CT without any symptom, symptomatic LI defined as opacities on chest X-ray and/or CT in association with hemoptysis and hypoxemia, renal dysfunction, allergic reactions, pulmonary artery perforation, rupture or dissection and periprocedural death (death during, or within 30 days of the procedure). The severity of LI was considered as mild (no treatment), moderate (requiring supplemental oxygenation) or severe (requiring mechanical ventilation and/or extracorporeal membrane oxygenation).

### 2.4. Statistical Analysis

Data are presented as mean ± standard deviation for continuous variables with normal distribution, and as median and interquartile range for non-normally distributed variables. Categorical variables were presented as frequencies and percentages (%). Differences in continuous variables such as hemodynamic parameters, were compared using the paired *t*-test for normally distributed variables and the Wilcoxon paired test for non-normally distributed variables. Categorical variables were expressed as number and percentage and were compared using the χ^2^ test for independence or Fisher’s exact test. The Kaplan–Meier method was used to estimate overall survival. For the survival analysis, the date of first BPA session was used as the start point to determine length of survival. The cut-off date was 10 February 2022. The Kaplan–Meier curve was supplemented by the proportion surviving at analyzed times. A *p*-value < 0.05 was considered statistically significant in this study. Data were analyzed using the SPSS version 16.0. (IBM SPSS Statistics for Windows, Version 16.0. Armonk, NY, USA)

### 2.5. Outcomes of BPA

The BPA procedure was defined as completed when all accessible lesions were addressed according to the current experience or if the patient did not consent to further treatment or for those with mPAP < 30 mmHg after BPA procedures.

For patients who were under specific PH medical therapy, drugs were interrupted if PH diagnosis according to current Guidelines [2] was not fulfilled after BPA procedures; specifically, if mPAP was equal to or lower than 25 mmHg and PVR was less than 3 WU.

The follow-up period for survival assessment ended on 10 February 2022. The primary endpoint for survival analysis was all-cause death. Long-term survival from the initial BPA procedure was assessed for all patients. The efficacy of BPA procedures was evaluated upon biochemical, clinical and hemodynamic parameters.

## 3. Results

### 3.1. Patient Characteristics

Up to 10 February 2022, 24 patients (79.2% females, mean age 53 ± 17 years (20–84) underwent BPA series. All 24 patients were referred to undergo BPA in a single referral center in Greece (there are two referral centers). Four patients were excluded from efficacy analysis because three of them had undergone only one or two sessions at time of cut-off date and the fourth died after an accident without having time to proceed with the BPA treatment, except for three sessions. Overall, 180 BPA sessions were conducted (2–17/patient, mean 8 ± 4/patient), and 1248 dilations were performed, 0–18 per session, 57 ± 36/patient. The predominant conditions associated with CTEPH were splenectomy and thrombophilic disorders. One patient received antipsychotic drugs which are associated with venous thrombosis [8,14].

All patients had been anticoagulated for at least 3 months. Anticoagulation consisted of direct oral anticoagulants in four patients (14.8%) and vitamin K antagonist in 23 patients (85.2%). At baseline, 87.5% of the study population was treated with home oxygen supplementation and 79.2% with PH specific drugs.

**Figure 2:**
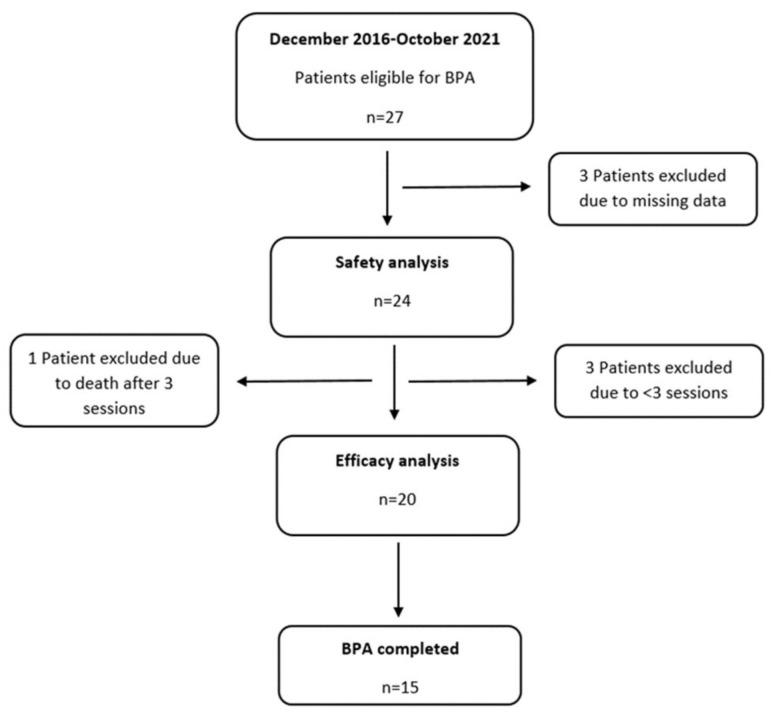
Flow chart of the study population. (The last patient included in the study underwent BPA in October 2021 and the Cut-off date for analysis is 10 February 2022).

Safety analysis was performed for all 24 patients (Figure 2).

For patients who had been previously operated on (*n* = 6), the median period from PEA to BPA was 74 months (IQR 114, range 13–272). For the remaining, the median time from diagnosis to the first BPA was 6 months (IQR 36, range 1–131).

Baseline characteristics of the overall population undergoing BPA (*n* = 24) are depicted in Table 1.

### 3.2. Effects of BPA

**Figure 3:**
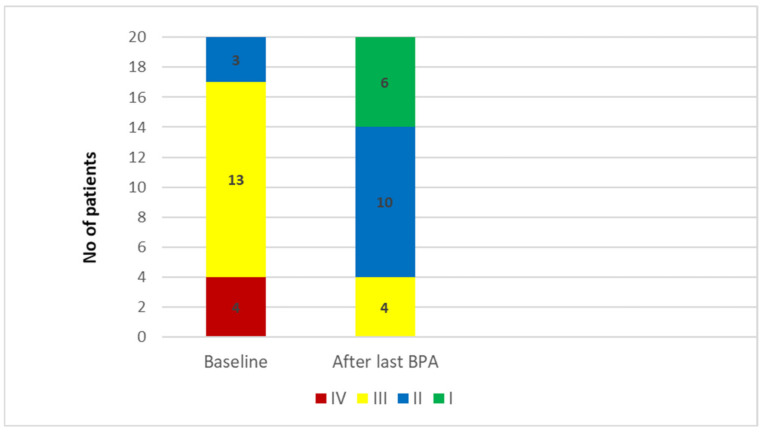
WHO (World Health Organization) functional class for the 20 patients assessed for Balloon Pulmonary Angioplasty efficacy.

In the 20 patients who underwent a completed clinical and hemodynamic evaluation after the last BPA, a significant improvement in WHO functional class, arterial and mixed venous saturation and hemodynamic parameters, except of CI, was observed. The percentage decreases in mPAP and PVR were 44% and 60% respectively; NT-proBNP level decreased by >70% compared to baseline value. In two patients with a history of PEA and lobar total occlusion, BPA failed to meet treatment goals. Table 2 shows the results of efficacy endpoints and Figure 3 the changes in WHO functional class.

After BPA, significantly fewer patients needed home oxygen therapy (85% vs. 35% for pre and post BPA respectively, *p* = 0.003), ameliorating quality of life. Furthermore, fewer patients continued to receive specific PH drugs and especially combination therapies. Efficacy data regarding post BPA CTEPH therapy are presented in Table 3.

Fifteen out of 20 patients are considered as having completed the BPA treatment and their results are depicted in Table 4.

### 3.3. Safety

Out of 180 BPA sessions, there was not a fatal complication. In total, 37 (20.6%) BPA-related non-fatal complications occurred in 10 (41.7%) patients. The percentage value of complications, reported in Table 5, is calculated assuming all complications even if a patient had two or three in the same session and in the same procedure. Fourteen patients had no complication. In our analysis, each complication was counted separately resulting in the sum of 37 (20.6%). But one complication, such as perforation, for instance, may have as a result hemoptysis and/or symptomatic lung injury counting another two complications. Complications occurred in 41.7% of patients and in 16.1% of all sessions, if calculated as complications in different sessions or patients, as other registries or series analyze.

All perforations resulted in hemoptysis. The one case of symptomatic lung injury was treated with NIPPV and the other case of symptomatic lung injury also had hemoptysis and was treated with NIPPV. We predominantly detected hemoptysis (*n* = 18, 10%) in six (25%) patients and only two patients were treated with non-invasive positive pressure ventilation (NIPPV) (1.1%). In three cases of hemoptysis (18.8%) there was a need for a sponge embolization of the culprit vessel. The other cases were self-limited with no action taken or resolved with prolonged balloon inflation proximal to the damaged site. Complications per session and per patients are summarized in Table 5.

The median duration of hospitalization was 1.3 days/patient. Mean serum creatinine levels did not change after the BPA series (82.8 ± 19.1 µmol/L before the first BPA and 84.3 ± 20.9 µmol/L after the last BPA).

### 3.4. Survival

In total, there were five deaths among 24 patients without a periprocedural death. The causes were as follows: one due to an accident and subsequent pulmonary embolism, one due to sepsis, one due to COVID-19 infection, one due to Parkinson disease and one death due to right heart failure 36 months after the last BPA procedure. No patient underwent lung transplantation.

**Figure 4:**
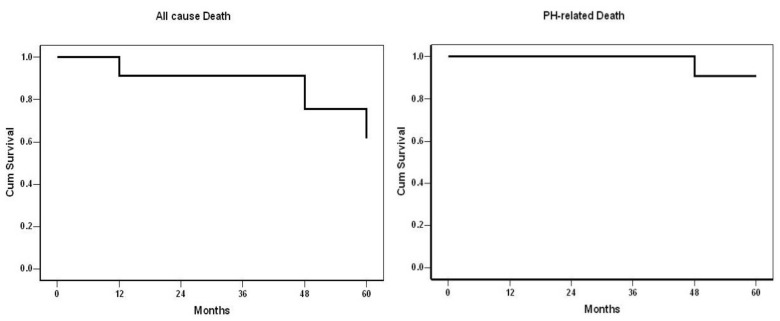
Overall survival of all 24 CTEPH patients treated with Balloon Pulmonary Angioplasty.

Overall survival at 1, 2, 3, 4 and 5 years was 91%, 91%, 91%, 75% and 62% respectively (Figure 4). The probability of not dying from PH was 100% for the first 3 years and 91% for the next two years.

## 4. Discussion

This registry represents the first description of BPA procedures in CTEPH patients in Greece. We demonstrated the favorable safety in 24 patients who underwent 180 BPA sessions. Moreover, we confirmed the efficacy of BPA with significant improvements in clinical and hemodynamic parameters. Likewise, patients had a significant decrease of NT-proBNP after the procedures and an impressive decreased use of supplemental oxygen therapy.

PEA is the treatment of choice for CTEPH patients; however, BPA is a very promising invasive non-surgical strategy of CTEPH treatment since even in specialized CTEPH centers up to 50% of patients diagnosed with CTEPH cannot undergo PEA [15] (as being inoperable or refusing surgery). Patients who do not undergo surgery have a deteriorated prognosis. Similarly, in approximately 25% of patients after PEA, PH remains associated with symptoms and a poor survival [15]. In the General University Hospital in Prague, according to the Czech registry [16], 42.2% of CTEPH patients do not undergo PEA and 30% of patients after PEA have residual PH. According to the worldwide prospective CTEPH Registry [17], 64.3% of recruited patients were eligible for PEA and 2% were candidates for both PEA and BPA, but only 58.7% of the entire population (1010 patients) underwent PEA.

**Figure 5:**
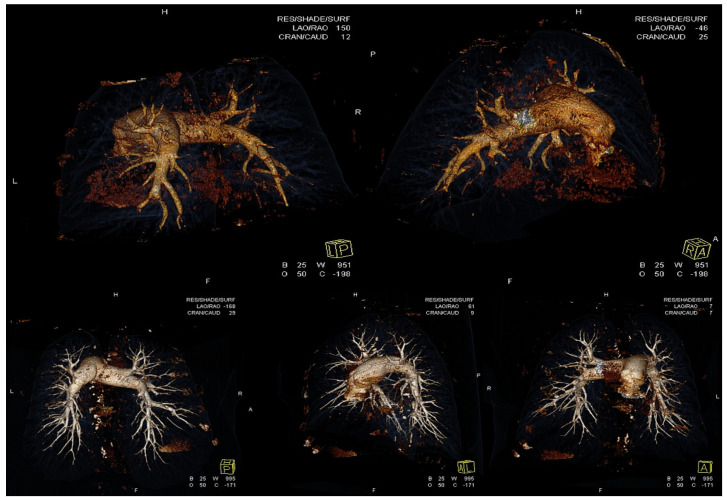
The improvement of anatomy in the pulmonary arterial tree via a Computed Tomography Angiography/3-D reconstruction study in a patient treated with BPA. Upper panel shows the pulmonary artery tree before BPA. Right and left main pulmonary arteries where some lobar and segmental branches are visible but without subsegmental branches. Lower panel shows the pulmonary artery tree after BPA where the majority of segmental and subsegmental branches are recanalized.

Pulmonary vascular resistance and mPAP represent prognostic hemodynamic measures for CTEPH patients [18]. Furthermore, targeting a normal or near normal mPAP with a PH management strategy, we achieve the best outcome as the greater afterload lowering leads to a better improvement of right ventricular function [19]. The Japanese and European series do not report significant differences in baseline mPAP, with 43.2 ± 11.0 mmHg in the patients studied by Ogawa et al. [6] and 40 mmHg in patients reported by Inami et al. [12], and there are comparable data with a mean value of 40 ± 12 mmHg in the study by Olsson et al. [20] and 43.9 ± 9.5 mmHg in the French series [21]. Previous data in the literature had demonstrated an association of a high mPAP before BPA with more frequent complications [13,18,21]. By baseline hemodynamics, our cohort appears to be more compromised in comparison with all published series in the literature. The baseline value of mPAP in our study was 51.3 ± 12.6 mmHg. BPA had impressively good results as a decrease in mPAP of 44% and in PVR of 60% was achieved, while the French group [21] reported 26% and 43% respectively. A Japanese registry conducted in seven experienced BPA centers [11] reported the greater BPA efficacy with a reduction of mPAP by 47.9%. In many European centers, the reduction of mPAP is lower than 30% and according to the recently published worldwide registry data [17], a 41.5% reduction of PVR was noticed in Europe. In two sites in Germany, 266 BPA sessions were performed in 56 patients and a decrease of 18% in mPAP and 26% in PVR was achieved [20]. Interestingly, in our cohort, six patients achieved normal hemodynamics with a mean PAP lower than 25 mmHg. Similarly, the great decrease of NT-proBNP reveals the RV afterload reduction throughout BPA treatment strategy as all patients already treated with PAH specific drugs were on stable medications for at least 6 months. The reduction in mean PAP is positively correlated with the number of lesions treated with BPA [22]. In most Japanese centers [23,24,25], an approach of treating the entire pulmonary tree is used. A modern BPA concept of total revascularization, which dilates all lesions in each segmental pulmonary artery, is also used in patients treated with BPA in Greece. Perhaps, the magnitude of hemodynamic improvement observed in our study was also correlated with the treatment period and the number of procedures per patient which was slightly higher in our study with a median of seven. The median value in the Japanese registry [22] was 4. In Figure 5 we can see the improvement of anatomy in the pulmonary arterial tree via a Computed Tomography Angiography/3-D reconstruction study in a patient treated with BPA.

Considering the pathogenesis of CTEPH, the dead space (volume of breath that does not participate in gas exchange) and intrapulmonary shunt are assumed to be responsible for hypoxia in CTEPH [26]. Oxygenation occurs in capillary vessels, and CTEPH includes various types of micro-vessel vasculopathies. BPA can significantly improve oxygenation targeting not only in proximal vessels but predominantly in the distal pulmonary vascular bed. The dead space is significantly improved by PEA and BPA as recanalization of vessels distal to the obstructions take place, leading to amelioration of gas exchange and oxygenation. The improvement in arterial oxygenation in our patients proves the efficacy of BPA procedures via a prompt revascularization of the lungs as Figure 5 shows.

According to the European Society of Cardiology guidelines [2], inoperable patients and patients with persistent PH after PEA should receive PH drug therapy. In recent years, the BPA technique improved resulting in good results and fewer complications [11]; however, the question of PH specific drug use in patients undergoing BPA remains unanswered. A decision is necessary from the experienced interdisciplinary CTEPH team. In the study by Jansa et al. [16], 44% of patients with completed BPA, 38% of patients in the French cohort [20] and 28% of the patients in the Japanese registry [22] had not received any specific PH drug at least 3 months before BPA initiation. In our cohort, 79% of patients received PH-specific drug therapy before BPA program initiation—a large proportion of patients, perhaps due to difficulties encountered by the absence of a PEA center in Greece (long time from diagnosis to PEA evaluation abroad) [9,27]. Anticoagulation therapy is the gold standard of drug treatment for CTEPH patients. NOACs’ use is increasing, however oral vitamin K antagonists are recommended according to European Guidelines. In our study, we were not able to report differences in outcome in subgroups treated with oral vitamin K antagonist vs. NOACs, due to a small sample size.

BPA is an invasive procedure associated with complications such as pulmonary oedema, lung injury, pulmonary artery branch injury, pulmonary hemorrhage or pleural effusion [28]. According to the Japanese multicenter registry, the overall rate of BPA related complications was 36.3%. Pulmonary injury and hemoptysis were reported as the most common complications and severe complications presented in 5.5% of all patients [6].

Out of 180 BPA sessions, we did not observe a fatal complication and the rate of non-fatal BPA-related complications was 20.6%. Hemoptysis was the most frequent event (in 10% of all BPA sessions), results corresponding well with experiences reported from other European centers. Two sites from Germany [19] reported a rate of complications of 9.4% and a mortality rate of 1.8%. In the French cohort [21] the observed rate of LI of all sessions was 9.1% compared to 17.8% in the Japanese registry [11].

Despite the potential risk of renal function impairment due to contrast media administration during BPA interventions, no statistically significant differences in serum creatinine value from baseline were reported. Of interest, Kriechbaum et al. [29] described a slightly better renal function in patients with chronic renal disease, probably due to better pulmonary and systemic hemodynamics after BPA.

Untreated CTEPH is characterized by clinical deterioration and poor survival [30]. PEA can improve survival in operable patients. In the International Prospective Registry with 679 incident CTEPH patients, survival was 93% and 91% at 1 and 2 years for those after PEA and 88% and 79% in non-operated patients, respectively [31]. Residual PH was deemed to play significant role in increasing mortality. Therefore, BPA in specialized centers is intended for inoperable CTEPH patients and patients with residual PH after PEA. Overall survival in our cohort was 91%, 75% and 62% at 1–3, 4 and 5 years, respectively, and is comparable with the survival data after BPA from the Japanese registry (survival at 1 and 2 years was 93% and 91%). In the multicenter registry of the adult and pediatric Polish population [32], the overall 3-year survival of 156 patients who underwent BPA was 92.4%. In our series, no peri-procedural death occurred during the study.

Our study has several limitations. Firstly, the number of included patients was relatively small; however, the number of procedures is analogous with other registries on BPA published in the literature. Moreover, we would like to emphasize that all BPA procedures are performed by a PH expert interventional cardiologist in a referral center in Greece. The fact that in Greece this center offers an integrated BPA program gives a limited bias for efficacy and safety in our data. Secondly, as it is an interim analysis, not all patients had completed their revascularization and follow-up assessments at the time of this report. Thirdly, no control group was implemented, but this arises from the nature of a real-life design. Furthermore, the occurrence of asymptomatic LI may be higher than that reported in this study, as chest Computed Tomography, which is much more sensitive than a chest X-ray, was scarcely conducted. Despite these limitations, we add important data demonstrating the significant and complementary role of BPA in an experienced pulmonary hypertension referral center in Greece.

## 5. Conclusions

Balloon pulmonary angioplasty is an effective invasive strategy with acceptable safety, improving the survival rate for patients with inoperable or recurrent/persistent CTEPH after PEA, representing an emerging therapeutic option in the field of CTEPH.

## Figures and Tables

**Table 1:** Baseline Characteristics and Hemodynamic Data. Variable *n* = 24.

Age (Years)	53 ± 17
Females (*n*, %)	19 (79.2)
WHO FC I/II/III/IV (%)	0/12.5/62.5/25
NTproBNP (pg/mL)	669 (IQR 153–1938)
Previous PEA (*n*, %)	6 (25)
Haemodynamics	
Systolic PAP (mmHg)	84.5 ± 23.5
Mean PAP (mmHg)	51.3 ± 12.6
Mean RAP (mmHg)	10.7 ± 4.0
PCWP (mmHg)	11.0 ± 2.7
CI (L/min/m^2^)	2.30 ± 0.64
PVR (WU)	10.66 ± 4.59
SaO2 (%)	89.6 ± 4.6
SvO2 (%)	60.9 ± 8.3
Heart Rate	82 ± 9
PH therapy	
ERA (*n*, %)	9 (37.5)
PDE5-I (*n*, %)	1 (4.2)
sGC stimulator (*n*, %)	14 (58.3)
Oral IP receptor agonist (*n*, %)	1 (4.2)
IV epoprostenol	1 (4.2)
Sc treprostinil	2 (8.3)
Inhaled Iloprost	1 (4.2)
None/single/double/triple (%)	20.8/45.8/25.0/8.3
Home Oxygen (*n*, %)	21 (87.5)
Associated Conditions	
Splenectomy (*n*, %)	6 (25)
Hemoglobinopathies (*n*, %)	4 (16.7)
Myeloproliferative disorder (*n*, %)	2 (8.3)
Thrombophilic disorder (*n*, %)	5 (20.8)
V/A shunt/Pacemaker (*n*, %)	2 (8.3)
APS (*n*, %)	2 (8.3)
Psychiatric disorder (*n*, %)	1 (4.2.)

WHO FC: World Health Organization Functional Class, PEA: Pulmonary Endarterectomy, PAP: Pulmonary Arterial Pressure, PCWP: Pulmonary Capillary Wedge Pressure, CI: Cardiac Index, PVR: Pulmonary Vascular Resistance, SaO2: Arterial Oxygen Saturation, SvO2: Mixed Venous Oxygen Saturation, PH: Pulmonary Hypertension, ERA: Endothelin Receptor Antagonist, PDE5-I: I: Phosphodiesterase-5 inhibitor, sGC: soluble Guanylate cyclase, IV: intravenous, Sc: Subcutaneous, APS: Antiphospholipid Syndrome, V/A: Ventriculoatrial.

**Table 2:** Clinical and Hemodynamic Data before and after last BPA (*n* = 20).

	Baseline	After BPA	Change (%)	*p*-Value
RAP (mmHg)	10.4 ± 4.0	6.5 ± 3.0	−37	<0.001
Systolic PAP (mmHg)	82.8 ± 25.3	44.6 ± 13.2	−46	<0.001
mean PAP (mmHg)	50.8 ± 13.5	28.6 ± 8.0	−44	<0.001
PVR (WU)	10.6 ± 4.9	4.2 ± 2.7	−60	<0.001
CI (L/min/m^2^)	2.32 ± 0.68	2.56 ± 0.44	+9	0.119
HR	84 ± 9	69 ± 11	−18	<0.001
SAO2	90.3 ± 4.8	95.5 ± 3.6	+5	<0.001
SVO2	62.4 ± 8.0	67.0 ± 6.6	+7	0.003
NTproBNP (pg/mL) (median)	912 ± 1158 (356)	220 ± 303 (91)	−76 (−74)	0.003
WHO FC (mean) (median)	3.05 ± 0.6 3	1.90 ± 0.7 2	−38	<0.001
WHO FC I/II/III/IV (%)	0/15/65/20	30/50/20/0		

BPA: Balloon Pulmonary Angioplasty, RAP: Right Atrial Pressure, PAP: Pulmonary Arterial Pressure, PVR: Pulmonary Vascular Resistance, WU: Wood Units, CI: Cardiac Index, HR: Heart Rate, SAO2: Arterial Oxygen Saturation, SVO2: Mixed Venous Oxygen Saturation, WHO FC: World Health Organization Functional Class, Functional Class.

**Table 3:** Treatment before and after the last Balloon Pulmonary Angioplasty (*n* = 20).

	Before BPA	After BPA	*p*-Value
Home oxygen therapy	17 (85)	7 (35)	0.003
PH specific drugs	15 (75)	11 (55)	0.320
Parenteral Prostanoids	3 (15)	2 (10)	0.002
Monotherapy	7 (35)	7 (35)	
Dual combination therapy	6 (30)	3 (15)	
Triple combination therapy	2 (10)	1 (5)	

PH: Pulmonary Hypertension, BPA: Balloon Pulmonary Angioplasty.

**Table 4:** Clinical and hemodynamic data of 15 patients who completed BPA treatment.

	Baseline	After BPA	Change (%)	*p*-Value
mRAP (mmHg)	10.0 ± 4.2	6.6 ± 2.9	−34	<0.001
Systolic PAP (mmHg)	76.8 ± 23.7	40.6 ± 11.9	−47	<0.001
mean PAP (mmHg)	47.8 ± 13.5	26.4 ± 7.6	−45	<0.001
PVR (WU)	10.0 ± 5.0	3.7 ± 2.8	−63	<0.001
CI (L/min/m^2^)	2.23 ± 0.7	2.51 ± 0.36	+11	0.143
HR	84 ± 10	68 ± 12	−19	<0.001
SAO2	90.4 ± 5.1	95.3 ± 4.0	+5	0.001
SVO2	63.4 ± 8.2	67.3 ± 7.1	+6	0.028
NTproBNP (pg/mL) (median)	925 ± 1238 (307)	231 ± 327 (81)	−75 (−74)	0.012
WHO FC (mean) (median)	3.0 ± 0.7 3	1.9 ± 0.7 2	−37	<0.001
WHO FC I/II/III/IV (%)	0/20/53.3/26.7	26.7/53.3/20/0		
Home Oxygen Therapy (*n*, %)	13 (86.7)	5 (33.3)		0.001
Specific PH Treatment (*n*, %)	11 (73.3)	7 (46.7)		0.041

BPA: Balloon Pulmonary Angioplasty, mRAP: mean Right Atrial Pressure, PAP: Pulmonary Arterial Pressure, PVR: Pulmonary Vascular Resistance, CI: Cardiac Index, HR: Heart Rate, SAO2: Arterial Oxygen Saturation, SVO2: Mixed Venous Oxygen Saturation, NTproBNP: N-terminal pro Brain Natriuretic Peptide, WHO FC: World Health Organization Functional Class, PH: Pulmonary Hypertension.

**Table 5:** Complications per sessions and per patients (a patient could simultaneously have two different complications in a session such as pulmonary artery perforation and hemoptysis).

	Sessions (*n* = 180) (*n*, %)	Patients (*n* = 24) (*n*, %)
Overall complications	37 (20.6)	10 (41.7)
Asymptomatic Lung Injury	4 (2.2)	4 (16.7)
Symptomatic Lung Injury	2 (1.1)	2 (8.3)
Hemoptysis	18 (10)	6 (25)
Dissection	3 (1.7)	2 (8.3)
Pulmonary Artery perforation	5 (2.8)	4 (8.3)
Pulmonary Artery Rapture	0	0
Renal dysfunction	0	0
Allergic reaction (shock)	2 (1.1)	2 (16.6)
NIPPV	2 (1.1)	2 (16.6)
Intubation	0	0
ECMO	0	0
Death	0	0

NIPPV: Non Invasive Positive Pressure Ventilation, ECMO: extra corporeal membrane oxygenation.

## Data Availability

Not applicable.
